# Identification of heat shock protein 90 and other proteins as tumour antigens by serological screening of an ovarian carcinoma expression library

**DOI:** 10.1038/sj.bjc.6600439

**Published:** 2002-08-01

**Authors:** L-Y Luo, I Herrera, A Soosaipillai, E P Diamandis

**Affiliations:** Department of Pathology and Laboratory Medicine, Mount Sinai Hospital, Toronto, ON M5G 1X5, Canada; Department of Laboratory Medicine and Pathobiology, University of Toronto, 100 College Street, Toronto, ON M3G 1L5, Canada

**Keywords:** tumour antigens, ovarian cancer, serological analysis of recombinant cDNA expression libraries (SEREX), heat shock protein 90 (hsp90), autoantibodies, cancer autoimmunity

## Abstract

Serological screening of recombinant cDNA expression libraries has been widely used for the identification of tumour antigens in various cancer types. Identification of tumour antigens in ovarian cancer may facilitate the development of vaccine-based therapies and of disease biomarkers. The purpose of our investigation is to identify tumour antigens in ovarian cancer by using the serological analysis of recombinant cDNA expression libraries method. A recombinant ovarian carcinoma cDNA expression library was screened with ascites fluid, pooled from five ovarian cancer patients. Twelve tumour antigens encoded by known genes were isolated, including ribosomal protein S18, heat shock protein 90, JK-recombination signal binding protein, ribonucleoprotein H1, RAN binding protein 7, TG-interacting factor, eukaryotic translation initiation factor p40 subunit, human amyloid precursor protein-binding protein 1, ribosomal protein L8, CDC23, IQ motif containing GTPase activating protein 1, and ribosomal protein L3. Heat shock protein 90 was chosen for further investigation. The prevalence of hsp90 autoantibodies in ovarian cancer was determined with immunoassay. Sera from 22 normal females, 32 from ovarian cancer (22 stage III/IV, 10 stage I/II), 37 colorectal cancer, 13 breast cancer, 10 lung cancer, 20 benign gynaecologic diseases, and 10 benign breast lesions were screened. Seven (32%) stage III/IV ovarian cancer, 1 (10%) stage I/II ovarian cancer, 1 (3%) colorectal cancer, 1 (8%) breast cancer, and 1 (5%) benign gynaecologic disease sera were found to contain hsp90 autoantibodies. These data support the view that hsp90 autoantibodies are frequently found in late stage ovarian cancer. Hsp90 may, therefore, represent a novel biomarker for ovarian cancer and a candidate ovarian cancer vaccine target.

*British Journal of Cancer* (2002) **87**, 339–343. doi:10.1038/sj.bjc.6600439 www.bjcancer.com

© 2002 Cancer Research UK

## 

Aberrantly expressed or mutated cellular and cell surface proteins in human cancer can break self-tolerance and trigger immune responses. These proteins are known as tumour antigens (Boon and van der Bruggen, 1996). One example of tumour antigens is p53 (Angelopoulou *et al*, 1994; Soussi, 2000). To facilitate the identification of tumour antigens in cancer, serological analysis of recombinant cDNA expression libraries (SEREX) technology was developed (Sahin *et al*, 1995). In this technique, sera from cancer patients are used to screen recombinant cDNA expression libraries, constructed from tumour tissues. Tumour antigens in the libraries are isolated due to their immunoreactivities to the autoantibodies in the sera. So far, more than 1000 candidate tumour antigens in many types of cancer have been identified (Chen, 2000). Tumour antigens identified by SEREX or similar techniques have two major clinical applications. First, tumour antigens induce production of autoantibodies, found in the circulation. These autoantibodies could represent novel biomarkers for cancer diagnosis, prognosis, monitoring, and predicting response to chemotherapy. A number of tumour antigen-derived autoantibodies have already been widely used as biomarkers in various types of cancer, such as p53, c-erbB-2/HER2/neu and MUC1/CA15.3 (Angelopoulou *et al*, 1996, 1997; Angelopoulou and Diamandis, 1997; Molina *et al*, 1998; Duffy, 1999). Second, these tumour antigens can be used as targets for immunotherapy of cancer. The identification of tumour antigens with SEREX is based on the presence of IgG autoantibodies, the formation of which relies on CD4+ T lymphocytes (helper T cells). The identified tumour antigens, then, confirm presence of a repertoire of CD4+ cells which recognize such antigens. CD4+ T lymphocytes are known to play pivotal roles in anti-tumour responses (Frasca *et al*, 1998; Pardoll and Topalian, 1998). Thus, SEREX-defined tumour antigens could be used as therapeutic cancer vaccines to provoke antitumour immune responses.

Ovarian cancer is the most lethal gynecologic malignancy. Its poor survival is due to late diagnosis and ineffective treatments. When ovarian cancer is diagnosed, more than 75% of patients have stage III/IV disease, which has a five-year survival rate of less than 25%. Current management of ovarian cancer includes cytoreductive surgery followed by platinum-based chemotherapy. However, only a proportion of patients benefits from chemotherapy. So far, there is no biomarker available to help predict which patients will respond to chemotherapy in ovarian cancer. There is an urgent need for such biomarkers as well as of new therapeutic regimens. Cancer vaccines, aiming at clearing residual tumour after surgery, are now under investigation in ovarian cancer (Butts, 1999). Therefore, tumour antigens identified by SEREX could provide candidate targets for ovarian cancer vaccines. Here, we attempt to identify tumour antigens in ovarian cancer with SEREX. Our previous work with SEREX and ovarian cancer has led to the identification of a novel gene which is down-regulated in testicular cancer and has an isoform solely expressed in the central nervous system (Luo *et al*, 2001). We now report identification of 12 new candidate ovarian cancer antigens one of which, heat shock protein 90 (hsp90), was studied in detail and found to be immunogenic in 30% of advanced stage ovarian cancer.

## MATERIALS AND METHODS

### Immunoscreening of an ovarian carcinoma cDNA expression library with ascites fluid from ovarian cancer patients

The Uni-ZAP™ premade ovarian carcinoma cDNA expression library was purchased from Stratagene, La Jolla, CA, USA. Ovarian cancer ascites fluids were pooled from five different primary ovarian cancer patients. The cDNA library was plated on NZY agar plates at a density of 500 clones/15 cm plate. The plates were incubated at 42°C for 4 h to allow plaques to develop. Nitrocellulose filters soaked with isopropyl β-D-thiogalactopyranoside (IPTG) were then laid on top of the plaques and incubated at 37°C for 4 h to transfer the plaques onto the membranes. Subsequently, the filters were blocked with 5% non-fat dry milk/PBS-T (80 mM sodium orthophosphate, 20 mM sodium dihydrogen orthophosphate, 100 mM sodium chloride, and 0.1% Tween-20) overnight, at 4°C. To screen the library, the ascites were diluted 1 : 100 in 5% non-fat dry milk/PBS-T. The diluted ascites was first incubated with *E. coli* phage lysate (from Stratagene) for 2 h at room temperature to minimize the cross-reaction between the autoantibodies and the bacterial/phage proteins. Nitrocellulose filters were then incubated with this preabsorbed ascites for 2 h at room temperature to identify cellular proteins that react with the autoantibodies in the ascites. Following probing with the ascites, the filters were washed with PBS-T for three times and further treated with alkaline phosphatase-conjugated goat anti-human IgG (Jackson Immunoresearch Laboratories, West Grove, PA, USA) diluted 2000-fold in 5% non-fat dry milk/PBS-T for 1 h at room temperature. The filters were washed again as above and then proceeded to chemiluminescence detection with a dioxetane-based substrate (Diagnostic Products Corporation, Los Angeles, CA, USA). The plaques exhibiting immunoreactivities were excised from the plates and the phages were converted into pBluescript phagemids by *in vivo* excision with Exassist™ helper phage, following the manufacturer's instructions. The excised phagemids were purified and subjected to automated DNA sequencing with M13 forward and reverse primers. The insert sequences were compared to the known sequences in the Genbank database with the BLASTN alignment algorithm (Altschul *et al*, 1997).

### Identification of autoantibodies against heat shock protein 90 in various cancer sera with immunoassay

Purified heat shock protein 90, isolated from HeLa cells, was purchased from Stressgen, Victoria, BC, Canada. For the immunoassay, 100 μl/250 ng well^−1^ of hsp90 diluted in the coating buffer (0.1 M ethanolamine in phosphate buffer saline (PBS), pH 7.2) were first directly coated on the 96-well white polystyrene plate (Greiner Labtechnik, Germany). 100 μl well^−1^ of 6% bovine serum albumin (BSA) was also coated as a background control. After overnight incubation at room temperature, the plate was washed three times with washing buffer (containing 9 g l^−1^ NaCl and 0.5 g l^−1^ Tween 20 in 10 mmol l^−1^ Tris buffer, pH 7.40), then blocked with 200 μl well^−1^ coating buffer for 1 h. Subsequently, the plate was washed again as above. 100 μl of human sera, diluted 50-fold in assay buffer (containing 60 g l^−1^ BSA, 50 mmol l^−1^ Tris, pH 7.80, 2.5% normal mouse serum, 15% normal goat serum, 1% bovine IgG, and 0.5 g l^−1^ sodium azide) were then pipetted into both BSA and hsp90-coated wells, incubated for 1 h and washed six times. Finally, 100 μl well^−1^ of alkaline phosphatase-conjugated goat anti-human IgG, Fc fragment specific antibody (Jackson Immunoresearch, West Grove, PA, USA) diluted 5000-fold in assay buffer was added, incubated for 1 h and washed. The signal was detected with a time-resolved fluorometric procedure as follows: 100 μl of diflunisal phosphate substrate, diluted 10-fold in substrate buffer (0.1 M Tris, pH 9.1, 0.1 M NaCl and 1 mM MgCl_2_) were pipetted into each well and incubated for 10 min. 100 μl of developing solution (1 M Tris base, 0.4 M NaOH, 2 mM TbCl_3_, and 3 mM EDTA) were then added on top. The fluorescence was measured with a time-resolved fluorometer, the Cyberfluor 615 Immunoanalyzer (MDS Nordion, Kanata, ON, Canada). The details of this procedure have been published elsewhere (Christopoulos and Diamandis, 1992). All serum samples were analysed in duplicate.

### Patient sera

The samples used in this study are residual sera of routine testing from healthy blood donors and patients with various malignancies and benign diseases. We included sera from 22 normal females and the following patients: 32 ovarian cancer (22 stage III/IV, 10 stage I/II), 13 breast cancer, 10 prostate cancer, 10 lung cancer, 20 benign gynecologic diseases, and 10 benign breast lesions. All sera were stored at −80°C until analysis.

## RESULTS

### Isolation of tumour antigens by immunoscreening of an ovarian carcinoma cDNA expression library with ascites fluid from ovarian cancer patients

**Figure 1:**
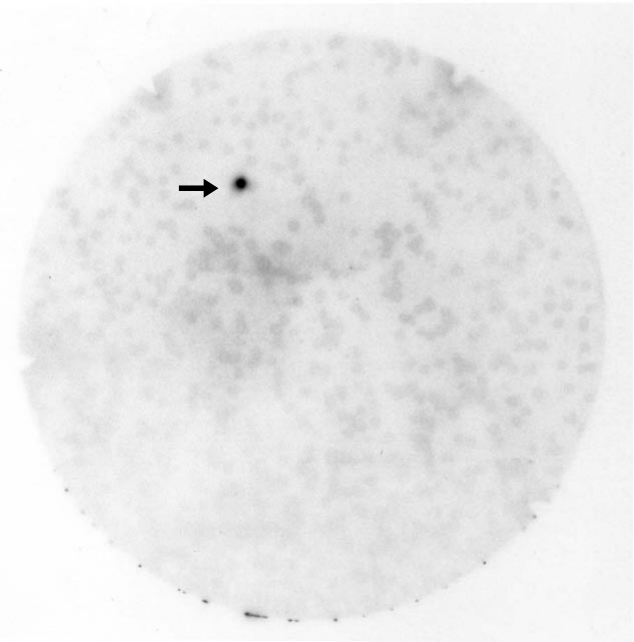
An example showing a positive clone identified by immunoscreening of an ovarian carcinoma cDNA expression library. The black dot indicated by an arrow is the positive clone.

A recombinant ovarian carcinoma cDNA library was screened with ascites pooled from five ovarian cancer patients. In total, 50 000 clones were screened. Twelve clones were found to have immunoreactivities. An example of a positive clone is shown in Figure 1

. In order to confirm the identities of these positive clones, they were isolated and converted to phagemids. The inserts were then sequenced and compared to the Genbank database with the BLASTN program. All 12 positive clones are known proteins (Table 1

**Table 1:**
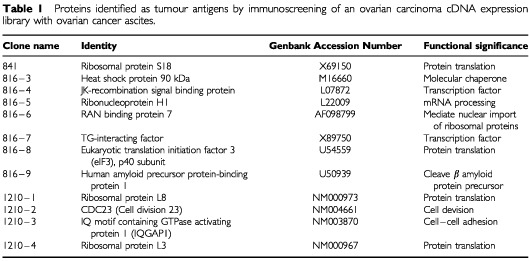
Proteins identified as tumour antigens by immunoscreening of an ovarian carcinoma cDNA expression library with ovarian cancer ascites

). Most of these proteins are involved in gene transcription, protein translation, and mitosis. Heat shock protein 90 was selected for further evaluation because this protein is commercially available in pure form, allowing development of an ELISA-type screening assay.

### Determination of the prevalence of heat shock protein 90 autoantibodies in ovarian cancer

SEREX technology identifies tumour antigens by screening with sera which are usually obtained from a small number of patients. The immunogenicities of the identified tumour antigens should therefore be tested in a larger patient population, to determine their prevalence in various types of cancer. Since the proteins expressed in the prokaryotic recombinant cDNA expression libraries may have different conformation or post-translational modifications compared to their native human forms, the autoantibodies could recognize the recombinant proteins, but not the native forms. Hsp90 was chosen for further evaluation because it was the only commercially available antigen from the list of Table 1. Purified human hsp90 directly coated on the microtiter plate was used to examine the presence of autoantibodies in the sera from normal controls, various cancers, and benign diseases. In total, sera from 22 normal female, 32 ovarian cancer (22 stage III/IV, 10 stage I/II), 37 colorectal cancer, 13 breast cancer, 10 lung cancer, 20 benign gynecological diseases, and 10 benign breast lesions were screened. For the 22 normal sera, fluorescence count ratios between hsp90-coated wells *vs* BSA-coated wells had a mean of 1.42 and a standard deviation of 0.28. Thus, the upper limit of the fluorescence ratio was selected as 2.00, which represents the mean plus two standard deviations (95% percentile). Sera with ratios >2.00 were defined as hsp90 autoantibody-positive. With this method, 7 (32%) stage III/IV ovarian cancer, 1 (10%) stage I/II ovarian cancer, 1 (3%) colorectal cancer, 1 (8%) breast cancer, and 1 (5%) benign gynecological disease sera were found to contain hsp90 autoantibodies (Table 2

**Table 2:**
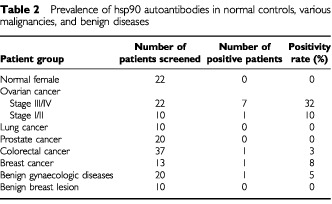
Prevalence of hsp90 autoantibodies in normal controls, various malignancies, and benign diseases

). Antibody titers for all sera tested are shown in Figure 2

**Figure 2:**
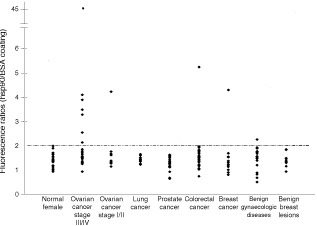
Distribution of hsp90 autoantibodies in sera from normal females, patients with various types of cancer and benign diseases. The dotted line indicates the cutoff, 2.0.

. At the level of 100% specificity (fluorescence ratio >2.3), there are still six sera from late stage ovarian cancer, one serum from early ovarian cancer, one serum from colorectal cancer, and one serum from a patient with breast cancer are positive for hsp90 autoantibodies. Our results indicate that hsp90 autoantibodies are mostly present in late stage ovarian cancer.

## DISCUSSION

In our immunoscreening of the ovarian carcinoma cDNA expression library, twelve known proteins were identified as putative tumour antigens (Table 1). To the best of our knowledge, these proteins have not been previously reported as ovarian tumour antigens. However, some of these proteins or their homologues have already been recognized as tumour antigens by SEREX in other types of cancer, such as the JK recombination signal binding protein, eIF2B (a homologue of eIF3) (Gure *et al*, 2000), and hsp70 (a homologue of hsp90) (Scanlan *et al*, 1998). One feature of many proteins that we identified is their involvement in gene transcription, protein translation, and mitosis. Among the 12 proteins of Table 1, 10 belong to these categories. This pattern is consistent with the SEREX-defined tumour antigens reported in the literature (Chen, 2000). Although it seems that the immune responses to these housekeeping proteins may be nonspecific and likely related to the high turnover and necrosis associated with cancer, new experimental evidence indicates that these immunogenicities actually result from specific structural changes or aberrant expression of these proteins. For example, CDC27, which participates in mitosis, is an MHC class II-restricted tumour antigen in melanoma. It has been shown that a mutation within this gene causes protein structure change, and hence, induction of immune responses (Wang *et al*, 1999). In addition, eukaryotic translation initiation factor 4 (eIF4), which is involved in protein translation, is a SEREX-defined antigen in lung cancer. It has been shown that its immunogenicity is due to overexpression (Brass *et al*, 1997). We speculate that the proteins identified here generate autoimmune reactions through similar mechanisms. It is noteworthy that eIF3 and CDC23, which are the homologues of eIF4 and CDC27, respectively, were identified in our screening. Some of the proteins that we identified have already been reported to be overexpressed in various types of cancer, including ribosomal protein S18 (Chassin *et al*, 1994), RAN binding protein 7 (Li *et al*, 2000), and eIF3, p40 subunit (Nupponen *et al*, 1999). Therefore, given the aberrant expression of these proteins in various malignancies, it is not surprising that they elicit immune responses.

To examine the prevalence of the hsp90 autoantibodies in ovarian cancer, purified human hsp90 was used in an ELISA-type screening assay. In our initial SEREX library screening, we utilized ascites fluid pooled from five ovarian cancer patients with late stage disease. Hsp90 autoantibodies have the highest frequency (32%) in late stage ovarian cancer, which is consistent with the presence of hsp90 autoantibodies in the initial ascites pool. Hsp90 autoantibodies were also observed in colorectal cancer (3%) and breast cancer (8%). It appears that hsp90 is a tumour antigen shared by various types of cancer. The correlation of hsp90 autoantibodies and late stage ovarian cancer implies that it may have utilities as a novel prognostic biomarker for ovarian cancer. In osteosarcoma, hsp90 autoantibody positivity has been found to be associated with response to chemotherapy, whereas, its absence correlates with metastasis (Trieb *et al*, 2000). In contrast, Conroy *et al* (1998) reported that the presence of hsp90 autoantibodies indicated metastasis in breast cancer. What hsp90 autoantibodies could predict in ovarian cancer awaits further detail investigations to analyse the relationship of hsp90 autoantibodies and various clinicopathological variables.

Hsp90 has been investigated as a cancer vaccine target in animal models. It has been shown that hsp90 purified from autologous tumours has a protective effect in mouse (Graner *et al*, 2000; Ullrich *et al*, 1986). It is now believed that such a protective effect of hsp90 is mainly due to the tumour-derived antigenic peptides that bind to it (Ishii *et al*, 1999; Srivastava *et al*, 1998; Wells and Malkovsky, 2000). In our study, since the prokaryotic expression library and purified hsp90 from HeLa cells are unlikely to harbour such antigenic peptides, it seems that the immunoreactivities that we observed are hsp90 specific. Our results indicate that hsp90 alone could be used as a cancer vaccine target.

In summary, we show here that many candidate tumour antigens could be identified in SEREX screening of ovarian cancer. Our findings warrant further investigations on these proteins, aiming to elucidate their immunogenicity in ovarian cancer. Hsp90 is immunogenic in 32% of late-stage ovarian cancer, suggesting that it is a potential target for a therapeutic vaccine in ovarian cancer.
